# Risk factors of unintended return to the operating room in adult spinal deformity

**DOI:** 10.1186/s13018-021-02385-7

**Published:** 2021-04-06

**Authors:** Lung Chan, Yue Li, Yong Hai, Yuzeng Liu, Yangpu Zhang

**Affiliations:** Department of Orthopedic Surgery, Beijing Chaoyang Hospital, Capital Medical University, No. 8 Gong Ti Nan Lu, Chaoyang District, Beijing, People’s Republic of China 100020

**Keywords:** Adult spinal deformity, Unintended return to OR, Apical vertebral rotation, Neurologic deficit, Implant malposition

## Abstract

**Background:**

To evaluate the incidence and risk factors associated with unintended return to the operating room in adult spinal deformity after spinal deformity corrective surgery.

**Methods:**

Retrospect of 141 adult spinal deformity patients in a single institution between January 2017 and December 2019. Inclusion criteria enrolled 18 to 80 years old patients who diagnosed with congenital/idiopathic/syndromic/acquired spinal deformity underwent posterior corrective spinal surgery. The surgical details and complications were recorded. The rate of unintended return to the operating room (UIROR) during hospitalization was examined, and the risk factors of unintended return to the operating room were investigated via multivariate analysis.

**Results:**

This is a retrospective study. One hundred and forty-one patients who underwent spinal deformity surgery with a mean age of 31.8 years (range 18-69 years) were studied. The rate of unintended return to the operating room was 10.64% (15/141). Two of 15 patients had twice unintended surgery during hospitalization (13.33%). The most principal complication was neurologic deficit (73.3%); six of 15 postoperative present implants deviation causes severe lower limbs radiating pain (40%). The multivariate analysis shows higher apical vertebral rotation (AVR>grade II, odds ratio [OR] = 9.362; 95% CI= 1.930-45.420; *P*= .006), obesity (OR = 11.448; 95% CI= 1.320-99.263; *P*= .027), and previous neurological symptom (OR = 7.358; 95% CI= 1.798-30.108; *P*= .006) were independent predictors of unintended return to the operating room.

**Conclusion:**

Postoperative neurologic deficit and short-term implant malposition are essential causes of unintended return to the operating room in adult spinal deformity patients. Preoperative factors such as higher AVR (> grade II), obesity, and previous neurological symptom may significantly increase the risk of morbidity in UIROR. Spine surgeons should be alert to these risk factors and require adequate preoperative evaluations to reduce the incidence of unintended return to the operating room.

## Introduction

Adult spinal deformity (ASD) includes not only the coronal and sagittal deformities but also rotational subluxation and axial plane deformity, is an increasing public health concern, people of any age and gender may suffer from spinal deformity [1, 2]. ASD may develop from congenital, idiopathic spinal deformities in childhood and adolescence, or due to degenerative changes in intervertebral disks and facet joints [3–6]. ASD may also be the result of trauma, tumor, infection, or inflammation affecting the spine. According to reports, the prevalence of ASD in the general population is as high as 32% [5, 6], ASD patients may experience symptoms related to pain, the progression of deformity, coronal or sagittal malalignment, and/or neurologic deficit [7].

Corrective surgery for ASD is a complex procedure that aims to reduce pain, disability, the progression of the deformity, and improving function [8, 9]. Several authors reported ASD corrective surgery may be technically challenging, and adverse events often occur with a high rate of perioperative and postoperative complications (26.8% to 42%) [10–15]. In previous studies, complex ASD corrective surgery has been associated with a series of complications, such as wound infection, proximal or distal junctional failure, neurological deficits, and acute hemorrhagic anemia [16]. Some complications required surgical intervention in time after primary surgery. In the past 3 years, the complication rate in the author’s hospital was 27.6%; the most common cause was neurological deficit (14.9%), followed by wound infection (10.6%). A total of 38.4% of patients with complications need UIROR during hospitalization.

UIROR during hospitalization is an unexpected outcome, causing psychological, physical, and financial burden on patients with spinal deformity. Causes associated with UIROR include neurological complications, internal fixation deviations, and surgical site infections. This situation has become increasingly important in recent years. The Scoliosis Research Society Morbidity and Mortality Committee (SRS M&M) has enrolled the data of UIROR since 2017. Thus far, the SRS database has been used to report the M&M of idiopathic, congenital, neuromuscular, and other scoliosis and kyphosis. Therefore, the rate of UIROR after spinal corrective surgery cannot be underestimated. No previous reports on UIROR for patients undergoing spinal deformity surgery. Its risk factors remain undefined.

The goal of our research is to evaluate the incidence, causes, and risk factors associated with UIROR after ASD surgery.

## Materials and methods

This is a retrospective study. At the present study, we enrolled 141 consecutive patients who were diagnosed with ASD to evaluate radiologic and clinical outcomes between January 2017 and December 2019 and performed posterior instrumented corrective spinal surgery by 1 surgeon in a single institution. The patients were grouped based on whether they returned to OR unintended during hospitalization or not. This study was approved by the appropriate institutional review board of Beijing Chao-Yang Hospital. The authors are accountable for all aspects of the work in ensuring that questions related to the accuracy or integrity of any part of the work are appropriately investigated and resolved.

Included criteria are as follows: (1) Age 18 to 80 years old inclusive. (2) Congenital deformity (failure of formation and/or segmentation, kyphosis). (3) Idiopathic, syndromic or acquired scoliosis, kyphosis, or kyphoscoliosis. (4) Underwent corrective spinal surgery via the posterior approach.

Excluded criteria are as follows: (1) Underage or overage. (2) Patients with active infection. (3) With spinal tumor. (4) Underwent corrective spinal surgery via non-posterior approach (anterior or lateral). (5) Incomplete clinical data.

### Data collection

**Fig. 1:**
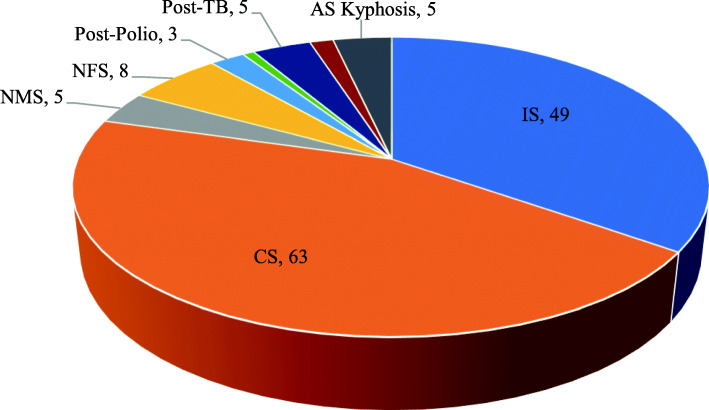
Diagnosis category in this study

Clinical and radiographic measurements were obtained through the clinical records preoperatively; postoperatively for the 141 patients who met the inclusion criteria, the following information (Table 1) was collected: demographic characteristics (age, gender, body mass index, duration of spinal deformity history), surgical characteristics (total operative time, intraoperative estimated blood loss, spinal osteotomy, American Society of Anesthesiologists Classification, total fusion levels, and curve correction rate), radiographic characteristics (Cobb angle of main curves, curve flexibility, and apical vertebral rotation). There were 49 idiopathic scoliosis, 63 congenital deformities, 8 neurofibromatosis scoliosis [NFS], 5 neuromuscular scoliosis [NMS], 5 post-tuberculotic deformity [PTBD], 5 ankylosing spondylitis kyphosis, 3 post-poliomyelitis scoliosis [PPS], 2 post-traumatic deformity [PTD], 1 Scheuermann’s kyphosis (Fig. 1). The main outcome measures examined were UIROR occurring during hospitalization.

**Table 1:** Baseline characteristics of patients recruited in the study

Characteristic	*n* (%) or mean ± SD
Age	31.80±12.51
Gender (male/female)	49/92
Body mass index (BMI)	22.23±3.99
Underweight	22 (15.6%)
Healthy weight	79 (56%)
Overweight	31 (22%)
Obesity	9 (6.4%)
Previous spine surgery (yes/no)	18/123
Number of fusion vertebrae	10.82±3.35
2-6	22 (15.6%)
7-12	66 (46.8%)
13+	53 (37.6%)
Mean operative time in min	291.67±85.99
Intraoperative estimated blood loss in ml	836.52±641.34
Osteotomy	89/52
Thoracoplasty	26/115
Fusion to sacrum	11/130

*BMI* body mass index

### Statistical analysis

IBM SPSS Statistics V18 x32 was used to perform all descriptive and comparative analyses. Univariate analyses were performed to examine the relationship between demographics/operative parameters and the two outcomes of interest by using *t* test or *x*^2^ test for data comparison. A multivariate logistical regression analysis was performed, adjusting for the duration of the spinal deformity history, obesity (BMI > 30kg/m^2^), previous lower extremity neurological symptoms, AVR (based on Nash-Moe grading system), and congenital deformity. Variables that showed a univariate association with UIROR with a *P* value less than 0.05 were included in the forward-stepwise selection model. All statistical comparisons were considered significant with a *P* value less than 0.05.

## Results

### Demographics of the study sample

A total of 141 consecutive patients met the inclusion criteria (Table 1). The mean ± standard deviation age was 31.8±12.51 years and 65.2% of the patients were female and a mean BMI of 22.2±3.98 (range 15.1 to 35.2). Thirty-one (22%) of the study population was defined as overweight, nine (6.4%) of the study population was defined as obesity. The most primary diagnoses included congenital deformity (44.7%) and adult idiopathic scoliosis (34.8%). Eighteen (12.8%) of these patients had a previous spinal surgery history. The mean of total fusion levels were 10.82±3.35 with a range of 2 to 17 levels. Sixty-six (46.8%) of the patients underwent instrumented fusion of 7 to 12 vertebral segments. Eighty-nine (63.1%) of the patients underwent osteotomies, forty-three (48.3%) of 89 patients underwent grade 2 osteotomies, twenty-five (28.1%) patients underwent grade 5 or 6 osteotomies. The mean total operative time was 291.67±85.99 min, with a mean intraoperative estimated blood loss was 836.52±641.34 ml.

### Incidence rates and causes of UIROR

There were 15 patients (10.6%) who required UIROR during hospitalization. The median number of days after index corrective spinal surgery were 10.1 days (range 3 to 18). Two of 15 patients had twice unintended surgery during hospitalization (13.3%). The most common incident for UIROR was implants deviation causes severe lower limbs radiating pain (40%), followed by lower limbs paralysis (26.8%). Other indications included implants malposition found by computed tomography (CT) scan without any symptoms (20%), lower limbs paresthesia (6.6%), and wound infection (6.6%).

### Risk factors for UIROR

Fifteen UIROR patients and 126 non-UIROR patients with an average age of 34.47±14.95 (range 19–66) years and 31.48±12.22 (range 18–69) years, a mean BMI of UIROR were 25.05±4.93 kg/m^2^ (range 17.8–35.2) versus non-UIROR were 21.89±3.74 kg/m^2^ (range 15.1–34.9). Eleven (73.3%) of UIROR patients were diagnosed with congenital deformity. There was no significant difference in UIROR rate with any of the enrolled diagnoses. Patients in UIROR group with longer history of spinal deformity (26.2±15.32 years vs 18.9±12.6 years, *P* = .040). The factors that were significantly associated with UIROR are summarized in Table 2. The percentage of obese patients was significantly higher in the UIROR group (20.0% vs 4.8%, *P* = .046), as well as the percentage of patients with higher apical vertebral rotation (73.3% vs 37.3%, *P* = .011), and patients with preoperative neurological symptoms (66.7% vs 23.0%, *P* = .001). There was no statistically significant difference in other preoperative demographic data and imaging data between UIROR and non-UIROR group.

**Table 2:** Univariate analysis of UIROR risk factors

Parameters	UIROR	Non-UIROR	*P*
Patient-related			
Age (years)	34.47±14.95	31.48±12.22	.385
Gender (male/female)	7/8	42/84	.391
Obesity (obese/none)	3/12	6/120	.046*
Previous spine surgery history (yes/no)	0/15	18/108	.217
Duration of spinal deformity history (years)	26.20±15.32	18.86±12.62	.040*
Previous neurological symptom (yes/no)	10/5	29/97	.001*
Congenital deformity (yes/no)	11/4	52/74	.026*
Radiographic			
Preoperative Cobb angle of main curve (°)	91.61±30.34	89.62±36.09	.838
Preoperative flexibility of main curve (%)	16.20±16.98	14.37±14.55	.652
Preoperative maximum kyphosis angle (°)	77.56±37.87	72.23±43.32	.649
Preoperative AVR (>II/≤II)	11/4	47/79	.011*
Surgery related			
Total operative time (min)	317.00±110.01	288.65±82.71	.154
Intraoperative estimated blood loss (ml)	1060.00±701.83	809.92±631.51	.154
Number of fusion vertebrae	10.20±3.34	10.89±3.36	.454
ASA (>2/≤2)	1/14	33/93	.118
Osteotomy (yes/no)	10/5	79/47	.501
Thoracoplasty (yes/no)	3/12	23/103	.550
Fusion to sacrum (yes/no)	3/12	8/118	.096

*AVR* apical vertebral rotation, *ASA* American Society of Anesthesiologists

*P < 0.05, statistically significant difference between the two groups

The final multivariate regression (Table 3) for UIROR included duration of spinal deformity history, congenital deformity or not, higher apical vertebral rotation (AVR, OR = 9.362; 95% CI= 1.930-45.420; *P*= .006), obesity (OR = 11.448; 95% CI= 1.320-99.263; *P* = .027), and previous neurological symptom (*R* = 7.358; 95% CI= 1.798-30.108; *P* = .006).

**Table 3:** Multivariate analysis of UIROR risk factors

Parameters	*B*	SE	Ward	*df*	*P*	Exp(*B*)
Obesity	2.438	1.102	4.894	1	.027*	11.448
Duration of spinal deformity history	.017	.024	.513	1	.474	1.017
Previous neurological symptom	1.996	.719	7.707	1	.006*	7.358
Congenital deformity	−1.330	.725	3.370	1	.066	.264
AVR >II	2.237	.806	7.706	1	.006*	9.362

*AVR* apical vertebral rotation

*P < 0.05, statistically significant

## Discussion

ASD is an increasing public health concern, people of any age and gender may suffer from spinal deformity. Patients who undergo spinal corrective surgery may improve their quality of life. The rate of complications reported in the literature varies widely, ranging from 26.8 to 42% [10–15]. Urgent and severe complications may require UIROR. UIROR during hospitalization is an unexpected outcome, causing psychological, physical, and financial burden on patients with ASD. Nearly half of the UIROR patients because of postoperative severe lower limbs radiating pain caused by implants deviation.

As ASD patients grow older, the severity of the spinal deformity may also increase. Previous literature has proven that the incidence of complications in elderly patients has increased [17–19]. In this study, the age of the UIROR group was slightly older than that of the non-UIROR group, it did not reach statistical significance (34.47±14.95 years vs 31.48±12.2, *P* = .385). The duration of the history of spinal deformity in the UIROR group was significantly longer than that in the non-UIROR group (26.2±15.32 years vs 18.9±12.6 years, *P* = .040), but there was no significant statistical difference in multivariate analysis (*P* = .474). Some previous studies have reported the relationship between obesity and long-term outcomes and complications after ASD corrective surgery [20, 21]. Pull ter Gunne et al. [20] found that the incidence of wound infection in obese patients increased. It is speculated that the amount of subcutaneous fat that needs to be retracted, leading to more cell necrosis, and therefore the infection rate is higher. Similarly, Soroceanu et al. [21] found that obese patients had a higher rate of major complications and wound infections, but this did not affect the number of minor complications or the necessity of reoperation. In our case series, obese patients have a higher risk of UIROR during hospitalization with a statistical difference (OR = 11.448; *P* = .027).

As the predictor, preoperative high AVR (> grade II) were found to be significant risk factors in this study (OR = 9.362; *P* = .006). In the preoperative standing 36-in posteroanterior spine radiographs, eight of the patients with congenital deformity had high AVR, and the others were 2 patients with NFS and 1 with PPS. ASD may be longstanding and a stretch of evolvable deformity from primary disease, lending to increased scoliosis, kyphosis, and vertebral rotation. In the univariate analysis, the proportion of patients with congenital deformity in UIROR group was significantly more than in non-UIROR group (73.3% vs 41.3%, *P* = .026), but no statistical difference among the two groups (*P* = .066). There are no significant differences in the preoperative coronal and sagittal imaging parameters such as the cobb angle and flexibility of the main curve in our study (*P* > .05). We considered that the greater rotary vertebrae generally lead to angular torsion of the spinal cord, which increases the risk of postoperative neurologic complications, also increases the rate of UIROR. Future research should pay attention to this key point.

In terms of the surgical factors, the implant-related complications occurred in 9 patients (60%) of UIROR group, two-thirds of patients present implant-related low limb neurologic deficit. Soroceanu et al. [11] performed a multicenter, prospective study involving eleven institutions of 245 patients who underwent ASD surgery, 13.8% patients with implant-related complications, and more than half of them (52.6%) required reoperation within 2 years. Faloon et al. [22] compared the complications of primary and revision surgeries for 134 consecutive ASD patients treated with long fusions to the sacropelvis, the rate of return to the OR was 27.6%. In our study, three patients underwent spinal corrective and fusion from the thoracic spine to the sacrum in UIROR group, eight patients in non-UIROR group, without a significant statistical difference (20% vs 6.34% *P* = .096). Lee et al. [15] reported a National Surgical Quality Improvement Project (NSQIP) study based on 5803 patients, 150 (2.8%) patients unintended return to the OR due to short-term postoperative complications, the significant surgery-related predictors included long fusion (OR = 1.3, *P* = .002), posterior fusion (OR = 3.6, *P* < .0001), combined approach (OR = 3.3, *P* < .0001), pelvic fusion (OR = 1.9, *P* < .0001), osteotomy (OR = 2.1, *P* < .0001), and operative time >4 h (OR = 3.5, *P* < .0001). The above factors were not statistically significant in the univariate analysis in our series. However, there are differences between our study and the above literature, which may be caused by the different time points of the clinical observation results.

Postoperative neurological complication is one of the reasons for UIROR. In a multicenter, prospective, worldwide observational study, Lenke et al. [23] found a higher rate of postoperative neurologic deficit in patients with a preoperative neurologic deficit compared with patients without preoperative deficit (25.76% vs 22.17%, *P* < .0001). Kim et al. [24] reviewed 233 patients with ASD who underwent posterior vertebral resection, the preoperative neurologic deficit significantly increased complications (OR = 5.55, *P* = .0004). In this study, previous lower limbs neurological symptom is also an essential preoperative predictor (OR = 7.358; *P* = .006).

**Fig. 2:**
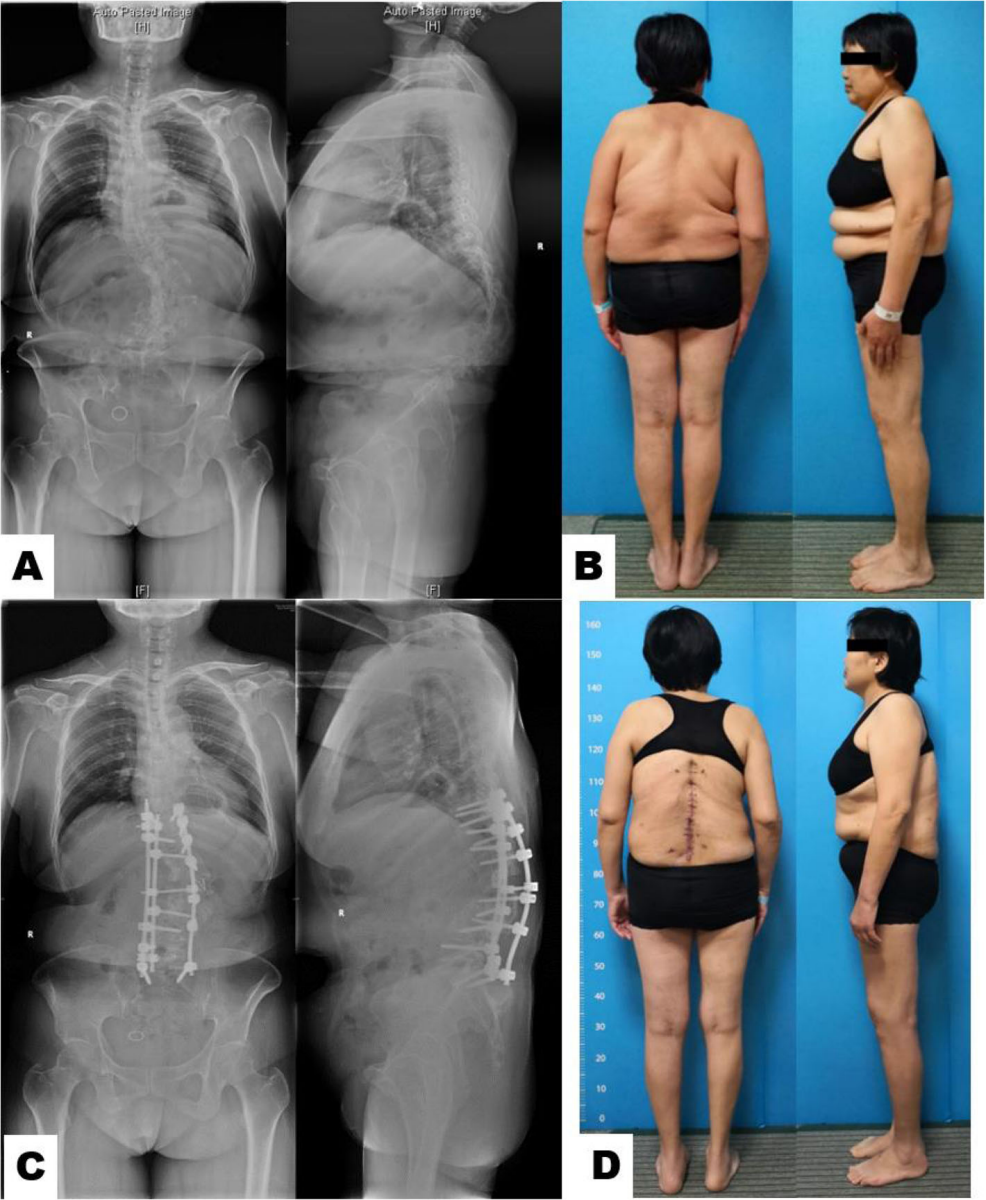
A 52-year-old woman of complex and rigid congenital kyphoscoliosis (L1/2 failure of segmentation) with obesity. She underwent spinal fusion from T9 to S1 and L1/L2 VCR. **a** Patients’ preoperative standing spine radiograph demonstrated the main curve of 88.3° and 92° thoracolumbar kyphosis with grade IV AVR. **b** Preoperative appearance photograph. **c** Postoperative standing spine radiograph showed the main curve was improved to 48°, with a correction rate of 45.6%. **d** Patients’ postoperative appearance photograph

The finding of the current study can be presented in the following case examples. Patient A (Fig. 2) was a 52-year-old woman with complex congenital kyphoscoliosis. She is obese (BMI=30.4). She had neurologic deficits for more than 20 years. Preoperative standing posteroanterior spine radiograph showed the AVR is grade IV. She underwent spinal fusion from T9 to S1 and L1/L2 vertebral column resection (VCR), with an operative duration of 320 min and estimated blood loss of 600 ml. On postoperative day 4, she developed severe left lower limb radiating pain, postoperative CT scan demonstrated that left L2 pedicle screw deviation. UIROR was performed on postoperative day 5. Patient A’s radiating pain was significantly relieved after implant adjustive and decompressive surgery.

There are still some limitations for our study. First, this is a retrospective single-institution study and thus the results may not be generalizable to other institutions. The relatively small sample size may have reduced the statistical significance to some extent, but all surgeries were performed by the same experienced surgeon and it shows predictors for UIROR in ASD surgery. We should expand the sample size in future work. Future study should consider the psychological, physical burden, and cost analysis which would improve our standing of the mental and financial impact of UIROR on ASD patients.

## Conclusion

In summary, the rate of UIROR after posterior corrective spinal surgery in this single institutional study was 10.6%. Postoperative neurologic deficit and short-term implant malposition are the main causes of UIROR. The risk factors of UIROR in ASD patients were preoperative AVR > grade II, obesity, and previous neurological symptom. Spine surgeons should be alert to these risk factors and require adequate preoperative evaluations for ASD patients to reduce the incidence of UIROR. This study can be used as an initial model for predicting UIROR in the study population.

## Data Availability

The data used and analyzed in this study is included in the article or are available from the corresponding and first authors on reasonable request.

## Abbreviations

UIROR: Unintended return to the operating room
ASD: Adult spinal deformity
AVR: Apical vertebral rotation
OR: Odds ratio
BMI: Body mass index
SRS M&M: Scoliosis Research Society Morbidity and Mortality Committee
NFS: Neurofibromatosis scoliosis
NMS: Neuromuscular scoliosis
PTBD: Post-tuberculotic deformity
PPS: Post-poliomyelitis scoliosis
PTD: Post-traumatic deformity
